# Books and Pamphlets Received

**Published:** 1884-05-20

**Authors:** 


### *BOOKS AND PAMPHLETS RECEIVED.*

*Elementary Principles of Electro-Therapeutics*, for the use of Physicians and Students, with one hundred and thirty-five illustrations, prepared by C. M. Haynes, M.D., published by the McIntosh Galvanic and Faradic Battery Co., Chicago, Ill. A work of four hundred and twenty-six large octavo pages, treating of *Magnetism*, *Franklinism*, *Galvanism*, *Electrolysis*, *Galvano-cautery*, *Faradism*, the McIntosh Combined *Galvanic Batteries*, *Electro-thermal Baths*, *Electro-Physiology*, *Electro-Diagnosis*, *Electro-Therapeutics*, etc.

Under these heads are Chapters which give detailed instruction of all important points connected with this interesting department of electro-therapeutics. Every Practitiouer would do well to procure and study this useful and interesting work.

*Sexual Neurasthenia, Nervous Exhaustion*—Its Hygiene, Causes, Symptoms and Treatment, with a Chapter on Diet for the Nervous. By George M. Beard, A. M., M. D., Formerly Lecturer on Nervous Diseases in the University of the City of New York; Fellow of the New York Academy of Medicine; Author of our "Home Physician;" "Hay Fever;" "Stimulants and Narcotics;" "Eating and Drinking," etc., etc. Posthumous M. S. Edited by A. D. Rockwell, A. M., M. D. Fellow of the New York Academy of Medicine; of the American Neurological Association and Electro-Therapeutist to the New York State Hospital; One of the Authors of "Medical and Surgical Electricity;" etc. New York: E. B. Treat, 757 Broadway. Price $2.00.

The above work will be read with interest, being upon subjects which, in years past, have been largely occupied by quacks or popular writers, solely in the interest of their pockets.

It is well that those affections connected with the abuse of the sexual organs should be investigated by men of true science, and that the regular Profession should be provided with books which will give information founded on science and truth, rather than on the assumption of base and irresponsible empirics.

*The Relation of Animal Diseases to the Public Health, and their Prevention.* By Frank S. Billings, D. V. S. Graduate of the Royal Veterinary Institute, of Berlin; Member of the Royal Veterinary Association of the Province of Brandenburg; Honorary Member of the Veterinary Society of Montreal, Canada, etc. New York: D. Appleton & Co., 1, 3 and 5 Bond street, 1884.

"It is the purpose," says the author, "to introduce to every thinking man and woman of the country, a new subject—the higher purposes of veterinary medicine. It is a work which treats of the prevention of diseases, not their treatment." The work is well timed, and will doubtless be read with interest, not only by medical men, but by intelligent men of the farming class. The fact that the diseases of animals have of late years been found to be analogous, and in many instances identical with those of the human species, makes the subject more attractive. The work before us has 441 pages and contains a vast amount of instructive and valuable reading.

*A. A. Mellier's Illustrated Catalogue* and Price List of Surgical Instruments and Appliances, St. Louis, Mo.

The above is a large Catalogue, containing one hundred and fifty pages octavo, with an extensive list of instruments of all kinds, Pocket and Surgical Cases, Syringes of every kind and variety, Laryngeal, Esophageal and Tracheal Instruments, Gynecological Instruments, Cupping, Aspirating and Dermatological, Electric, Orthopedic apparatus, Microscopes, Splints, etc., etc Address A. A. Mellier, 709 Washington Ave., St. Louis, Mo.

*A Treatise on Syphilis*, Its Rapid and Progressive Development among the Races, together with its Direful Effects upon the Human Family. Can it be Eradicated from the Human System by Medication? By James W. Price, M.D., Hot Springs, Arkansas.

*Iodoform in Dental Surgery*, by C. F. W. Bodecker, D.D.S., M.D.S., New York.

